# Small Extracellular Vesicles Promote Stiffness-mediated Metastasis

**DOI:** 10.1158/2767-9764.CRC-23-0431

**Published:** 2024-05-09

**Authors:** Alexandra Sneider, Ying Liu, Bartholomew Starich, Wenxuan Du, Praful R. Nair, Carolyn Marar, Najwa Faqih, Gabrielle E. Ciotti, Joo Ho Kim, Sejal Krishnan, Salma Ibrahim, Muna Igboko, Alexus Locke, Daniel M. Lewis, Hanna Hong, Michelle N. Karl, Raghav Vij, Gabriella C. Russo, Estibaliz Gómez-de-Mariscal, Mehran Habibi, Arrate Muñoz-Barrutia, Luo Gu, T.S. Karin Eisinger-Mathason, Denis Wirtz

**Affiliations:** 1 Department of Chemical and Biomolecular Engineering, Johns Hopkins Physical Sciences–Oncology Center and Institute for NanoBioTechnology, Johns Hopkins University, Baltimore, Maryland.; 2 Abramson Family Cancer Research Institute, Department of Pathology and Laboratory Medicine, Penn Sarcoma Program, University of Pennsylvania Perelman School of Medicine, Philadelphia, Pennsylvania.; 3 Johns Hopkins Institute for NanoBioTechnology, Johns Hopkins University, Baltimore, Maryland.; 4 Department of Materials Science and Engineering and Institute for NanoBioTechnology, Johns Hopkins University, Baltimore, Maryland.; 5 W. Harry Feinstone Department of Molecular Microbiology and Immunology, Johns Hopkins University Bloomberg School of Public Health, Baltimore, Maryland.; 6 Bioengineering and Aerospace Engineering Department, Universidad Carlos III de Madrid, Leganés, Spain.; 7 Instituto de Investigación Sanitaria Gregorio Marañón, Madrid, Spain.; 8 Johns Hopkins Breast Center, Johns Hopkins Bayview Medical Center, Baltimore, Maryland.; 9 Department of Oncology, Johns Hopkins School of Medicine, Baltimore, Maryland.

## Abstract

Tissue stiffness is a critical prognostic factor in breast cancer and is associated with metastatic progression. Here we show an alternative and complementary hypothesis of tumor progression whereby physiologic matrix stiffness affects the quantity and protein cargo of small extracellular vesicles (EV) produced by cancer cells, which in turn aid cancer cell dissemination. Primary patient breast tissue released by cancer cells on matrices that model human breast tumors (25 kPa; stiff EVs) feature increased adhesion molecule presentation (ITGα_2_β_1_, ITGα_6_β_4_, ITGα_6_β_1_, CD44) compared with EVs from softer normal tissue (0.5 kPa; soft EVs), which facilitates their binding to extracellular matrix proteins including collagen IV, and a 3-fold increase in homing ability to distant organs in mice. In a zebrafish xenograft model, stiff EVs aid cancer cell dissemination. Moreover, normal, resident lung fibroblasts treated with stiff and soft EVs change their gene expression profiles to adopt a cancer-associated fibroblast phenotype. These findings show that EV quantity, cargo, and function depend heavily on the mechanical properties of the extracellular microenvironment.

**Significance:**

Here we show that the quantity, cargo, and function of breast cancer–derived EVs vary with mechanical properties of the extracellular microenvironment.

## Introduction

The extracellular matrix (ECM), a network of acellular components predominantly made of collagen, controls tissue structure, modulates cell adhesion and dissemination, influences the secretome, and conveys mechanical signals (1–4). Tissues inherently have unique structures and stiffnesses that lend to specific biological processes (5–8). An increase in ECM stiffness often correlates with poor prognosis in solid tumors (9–14), explained in part by stiffness-mediated enhanced cancer cell migration and proliferation at the primary tumor (15–18). As tumors develop, the density and composition of the ECM changes (19). Because of chronic inflammation, often fibrotic tissue forms at and around the tumor site (20–22). The increased cross-linking of the ECM can lead to leaky vasculature and promote intravasation (23–26). Cellular phenotypes also change to promote tumor progression, that is, fibroblasts develop a cancer-associated phenotype that increases the deposition of fibrillar collagen and protumorigenic signaling (27, 28). Despite knowing the mechanical complexities of tumor growth and metastasis, cancer research and the development of therapeutics rely heavily on static model systems, such as tissue culture plastic, that do not incorporate physiologically relevant parameters (29, 30).

Because of their discovery, extracellular vesicles (EV) have primarily been collected and analyzed from tumor cells grown on tissue culture–treated plastic ware (31). Until recently, the physiologic relevance of EVs obtained in this manner remained largely unknown. Now EVs are a diverse group of lipid bilayer encapsulated particles secreted by cells that display and encapsulate functional proteins and nucleic acids (32, 33). Small EVs, particularly exosomes that are between 30 and 150 nm in diameter, have shown great promise as biomarkers and therapeutic agents for the treatment of disease (31, 34–37). Because of their size, exosomes have the potential to disseminate great distances from their site of secretion (38, 39). Small EVs can transfer their cargo to other cell types and influence homeostasis and disease progression (36, 40–46). Cancer-derived exosomes can increase vascular leakiness, reprogram bone marrow progenitors, increase tumor growth and metastasis (47), facilitate premetastatic niche formation (48–50), more effectively fuse with target cells (51); and aid in evading immune detection (31, 52). The role of tissue stiffness on EV secretion and metastasis remains largely unexplored (53, 54). Therefore, we sought to characterize the importance of physiologically relevant physical tissue properties (e.g., stiffness) in EV-mediated metastatic dissemination.

Herein we explore how modulating stiffness in the tumor microenvironment can influence cancer progression through EVs. We observed in primary patient tissues that more EVs are secreted from stiff tissue than softer tissue. We investigated exosomes isolated from breast cancer cells cultured on plastic (∼3 GPa), 25 kPa (breast tumor stiffness, stiff), and 0.5 kPa (normal tissue stiffness, soft) substrates. EVs from cells on substrates at tumor tissue stiffness have different cargo than those vesicles from soft and plastic substrates. The stiff EV cargo is enriched in integrins (ITGα_2_β_1_, ITGα_6_β_4_, ITGα_6_β_1_), adhesion proteins (CD44), and immune evasion signals over the soft and plastic EVs. These stiff EVs are better able to reach and be retained in distant tissues *in vivo* in mice and adhere to specific ECM proteins like collagen IV. In addition, stiff EVs promote cancer cell dissemination *in vivo* in zebrafish over soft EVs. EVs isolated from cells cultured on plastic do not consistently match either the physiologic stiff or soft conditions. Once cancer cells have arrived in distant tissues, the cells experience the mechanically soft environment of normal tissue. While stiff EVs appear to downregulate immune signaling from resident fibroblasts in the lung via a decrease in expression in *S100A4, S100A6, S100A12*, and *S100A13* to potentially to aid cancer cells in evading immune detection, the soft EVs demonstrate the ability to upregulate expression of cancer-associated fibroblast (CAF) markers (*ACTA2*, *COL1A1*, *VIM*) while also presenting increased inflammatory capacity (elevated *S100A10*, *S100A16*) in the resident fibroblasts. These results suggest that matrix stiffness influences vesicular secretion and cargo to aid cancer cells at different stages of the metastatic cascade.

## Materials and Methods

### Cell Culture

Human breast cancer cell lines MDA-MB-231 (RRID:CVCL_0062, Female) and human pancreatic cancer cell line BxPC-3 (RRID:CVCL_0186, Female) were obtained from ATCC. IMR-90 (RRID:CVCL_0347, Female) human lung fibroblasts were provided as a generous gift from Daniele Gilkes (Johns Hopkins University, Baltimore, MD). Cell lines were authenticated by initial vendor certification; annual authentication was not performed. All cell lines were cultured in DMEM (Corning, catalog no. 10-0130-CV) containing 10% FBS (Corning, catalog no. 35-010-CV) and 1% penicillin-streptomycin (Gibco, catalog no. 15140122; refs. 55, 56). Cells were maintained at 37°C and 5% CO_2_ and sustained in culture between passage 2 and 15. Cell lines were confirmed *Mycoplasma* negative using MycoAlert (Lonza, catalog no. LT07-318) every 6 months.

### EV Collection

Prior to vesicle collection, cells were seeded at 70% confluency and cultured on 177 cm^2^ 0.5 kPa Collagen Type I Coated Plates (Matrigen, catalog no. PS150-COL-0.5), 177 cm^2^ 25 kPa Collagen Type I Coated Plates (Matrigen, catalog no. PS150-COL-25), and 150 cm^2^ plastic tissue culture Falcon flasks (“plastic”; Corning, catalog no. 355001). After 24 hours incubation at 37°C, cells were washed with Dulbecco's phosphate buffered saline (DPBS, Corning, catalog no. 20-031-CV) and changed to DMEM containing 10% exosome-depleted FBS (Gibco, catalog no. A2720801) and 1% penicillin-streptomycin (Gibco, catalog no. 15140122). Following another 24 hours incubation at 37°C, EVs were collected and purified.

Cell culture supernatant was subjected to sequential centrifugation (800 × *g* for 5 minutes, 2,000 × *g* for 10 minutes, 10,000 × *g* for 30 minutes) at 4°C (Beckman Coulter Avanti J-E Centrifuge); filtered through a 0.22-µm PES filter (Genesee, catalog no. 25-244); and centrifuged twice at 100,000 × *g* for 2 hours at 4°C (Beckman Coulter Optima XE-90 Ultracentrifuge). Supernatant was replaced with DPBS between ultracentrifugation (UC) spins. The final vesicle pellet was resuspended in 1 mL DPBS. Samples were concentrated using a 2 mL 3 kDa Amicon filter (MilliporeSigma, catalog no. UFC200324). For the mouse experiments, samples were incubated with 1 µmol/L DiR dye (Thermo Fisher Scientific) prior to ultracentrifugation. For zebrafish and adhesion studies, EVs were labeled with the 7 µmol/L CMTPX Dye (Thermo Fisher Scientific, catalog no. C34552) between ultracentrifugation steps. Free dye was pelleted at 10,000 × *g* (3 × 10-minute spins) and EV containing supernatant ultracentrifuged and then concentration using Amicon. Protein concentration was determined using a Pierce bicinchoninic acid (BCA) protein assay kit (Thermo Fisher Scientific, catalog no. 23227) according to the manufacturer's protocol.

### Size Distributions of EVs

Size distribution and concentration of EVs were measured using a NanoSight NS300 (Malvern Preanalytical). Additional details are in the Supplementary Data.

### Western Blot Analysis

EV protein aliquots were lysed with 20% β-mercaptoethanol (Gibco, catalog no. 21985023) in 4X Laemmli buffer (Bio-Rad, catalog no. 1610747) for 5 minutes at 100°C. A total of 5 µg of EV protein lysate determined by BCA was loaded per lane and separated by molecular weight on 4%–15% Mini-Protean Precast TGX Gels (Bio-Rad, catalog no. 4561086) and transferred to Trans-Blot Turbo Mini polyvinylidene difluoride membranes (Bio-Rad, catalog no. 1620261). Overnight incubation at 4°C with primary antibodies, including anti-TSG101 (1:500, Abcam, catalog no. ab125011, RRID: AB_10974262) and anti-CD63 (1:1,000, Abcam, catalog no. ab193349, RRID: AB_3095976) in 1X Tris-buffered saline, 0.1% Tween (TBST). Secondary antibody incubation with corresponding horseradish peroxidase–conjugated anti-rabbit (Cell Signaling Technology, catalog no. 7074, RRID:AB_2099233) or anti-mouse secondary antibody (Cell Signaling Technology, catalog no. 7076, RRID:AB_330924).

### Silver Stain

Samples were prepared according to the Western blot protocol and run through a 4%–15% Mini-Protean Precast TGX Gel. The gels were then stained using the Pierce Silver Stain Kit (Thermo Fisher Scientific, catalog no. 24612) according to the manufacturer's protocol.

### Transmission Electron Microscopy

A total of 10 µL of sample was adsorbed to glow-discharged (EMS GloQube) 400 mesh ultra-thin carbon-coated grids (EMS CF400-CU-UL) for 2 minutes, followed by three quick rinses of TBS and stained with 1% UAT (uranyl acetate with 0.05% Tylose). Grids were immediately observed with a Philips CM120 at 80 kV and images captured with an AMT XR80 high-resolution (16-bit) 8 megapixel camera. Two biological repeats.

### Biodistribution of EVs in Mice

All mouse work was performed following Johns Hopkins University and International Animal Care and Use Committee (IACUC) guidelines under animal protocol MO16A383. There was no statistical method to predetermine sample size. Six to 8 weeks old NCr nude (NCRNU-F sp/sp) females (Taconic) were each injected via tail vein with stained vesicles in DPBS at a quantity of 10 µg of protein in 50 µL per mouse (56). Twenty-four hours after injection, mice and their organs were imaged using the LI-COR Pearl Impulse Imaging System (LI-COR Biosciences). Images were analyzed in LI-COR Pearl Impulse Imaging System according to the manufacturer's instructions. The mean signal-to-noise ratio (SNR) was determined by subtracting the mean background intensity from the mean intensity of the region of interest (ROI) and dividing through by the SD of the background.

### Biodistribution of EVs and Cancer Cells in Zebrafish

All procedures on zebrafish (Danio rerio) were approved by IACUC at The University of Pennsylvania (Philadelphia, PA). Fertilized zebrafish eggs of the transgenic strain expressing enhanced green fluorescent protein (EGFP) under the fli promoter (fli:EGFP) or mCherry under the flk promoter (flk:mCherry) were incubated at 28°C in E3 solution and raised using standard methods. Embryos were transferred to E3 solution containing 5 µg/mL proteases and 0.2 mmol/L1-phenyl-2-thio-urea (Sigma) 24 hours postfertilization to dechorionate the fish embryos and prevent pigmentation, respectively. At 48 hours postfertilization, zebrafish embryos were anesthetized with 0.03% tricaine (Sigma) and then transferred to an injection plate made with 1.5% agarose gel for microinjection. Approximately 200,000 EVs suspended in PBS were injected into the perivitelline space of each embryo using a XenoWorks Digital Microinjector (Sutter Instrument). Each injection volume was between 5 and 10 nL. At 2–2.5 hours after the vesicle injection, 150 to 400 MDA-MB-231 cells labeled with NucBlue live cell stain ReadyProbe (Thermo Fisher Scientific, catalog no. R37605) and suspended in complete growth medium supplemented with 0.5 mmol/L Ethylenediaminetetraacetic acid (EDTA) were injected into the perivitelline space of each vesicle-bearing embryo. Pre-pulled micropipettes were used for the microinjection (Tip inner diameter [ID] 50 µm, base outer diameter [OD] 1 mm, Fivephoton Biochemicals). After injection, the fish embryos were immediately transferred to a PTU-E3 solution. Injected embryos were kept at 33°C and were examined every day to monitor tumor cell migration using a widefield microscope.

### Patient Tissue Sample Preparation for Mechanical Measurements

All patient tissue samples were obtained with written consent from the patient and approved by the Johns Hopkins Medicine Institutional Review Board. Tissue samples received from the patients were kept in 4°C DPBS immediately after mastectomy or lumpectomy. Tumor samples were then transferred for mechanical tests within 4 hours of resection. The tumor tissue was then sectioned to expose the ROIs for micromechanical mapping and compression tests.

### Tumor Stiffness Mapping Using Microindentation

Dynamic indentation using a nanoindenter (Nanomechanics, Inc.) was used to characterize the tumor elastic modulus (57). Sneddon stiffness equation (58) was applied to relate dynamic stiffness of the contact to the elastic storage modulus of the samples (59, 60). Additional details are in the Supplementary Data document.

### Compression Tests of Tumor-adjacent and Tumor Tissues

Compression tests were performed as reported previously (61). Briefly, tissue samples were sectioned to obtain flat and parallel surfaces on all sides. Once the sample was sectioned, it was immediately staged on tensile/compression tester (MTS Criterion) for measurement (62). The top compression plate was lowered until in full contact with the tissue sample at minimal load. Once in contact, the samples could relax and stabilize for 1 minute before the actual compression test. Tissue samples were compressed at 0.25 mm/second deformation rate until 20% strain. Young modulus calculation was done on the best-fitted slope of the initial linear region (∼5%–10%) of the obtained stress-strain curve. A single measurement was obtained for each tissue.

### Vesicle Collection and Characterization for Patient Tissues

After mechanical measurements, tissue was transferred to 5 mL of 1% penicillin-streptomycin solution in 013-CV DMEM and incubated at 37°C overnight. After 24 hours, tissue was fixed in formalin and vesicles isolated from supernatant. Additional details are in the Supplementary Data document.

### ECM Binding Assay

Substrates were obtained from Millicoat ECM Screening Kit (MilliporeSigma, catalog no. ECM205) and rehydrated according to manufacturer specifications. A total of 1.5 × 10^9^ CMTPX fluorescently labelled vesicles in 50 µL DPBS (without Ca^2+^ and Mg^2+^) were incubated on each substrate for 1 hour at 37°C. Wells were imaged at 10X TRITC channel with 100% light intensity and 100 ms exposure time (Nikon Eclipse Ti), and fluorescence was determined in ImageJ (RRID:SCR_003070) via measuring the mean intensity of a fixed ROI. After removing the diluted suspension, the matrix was washed three times using DPBS (with Ca^2+^ and Mg^2+^; Corning, catalog no. 20-030-CV) according to the manufacturer protocol and the wells were imaged again under DPBS (without Ca^2+^ and Mg^2+^) to minimize possible fluorescence deviation from ions. For all washing steps, slow manual pipetting was adopted to avoid disturbance of the adhered EV samples. Background intensity was determined from the negative control—PBS with CMTPX dye processed through the same ultracentrifugation and 3 kDa Amicon filtration steps as EV samples—and subtracted from sample intensity. Sample intensity postwash was divided by the prewash intensity values at same ROI to determine percentage of vesicles adhered to each substrate.

### Fibroblast mRNA Expression Assay

IMR-90 lung fibroblasts were seeded 2 days prior to the addition of vesicles. These cells were then washed with DPBS and incubated in exosome-depleted medium with vesicles or DPBS for 48 hours at 37°C. RNA was extracted according to manufacturer instructions for DirectZol Kit (Zymo Research, catalog no. R2050) after imaging (Nikon Eclipse Ti). cDNA was generated using iScript cDNA Kit (Bio-Rad, catalog no. 1708890) according to manufacturer instructions. Then qPCR was performed. Two biological repeats with three technical repeats per condition. Housekeeping gene value is a geometric mean of α-tubulin (*TUBA3C*), *GAPDH*, and TATA-Box Binding Protein (*TBP*). The primer sequences used are listed in Supplementary Table S1.

### Quantifying EV Secretion

To determine quantifiable variations, nanoparticle tracking analysis (NTA) particle concentrations were multiplied by UC sample volumes for a total particle number. Dividing the total vesicle number by the weight of the tissue provides a value of vesicles secreted per gram of tissue.

### EV Proteomics

10-plex Tandem Mass Tag Proteomics (TMT Proteomics) performed on three biological replicates of EVs from cells cultured on tissue culture plastic, 25 kPa, and 0.5 kPa matrices (63). Data searched using SwissProt *Homo Sapiens* database with MASCOT in Proteome Discoverer 2.2 (RRID:SCR_014477). Additional details are in the Supplementary Data document. The mass spectrometry proteomics data have been deposited to the ProteomeXchange Consortium via the PRIDE [1] partner repository with the dataset identifier PXD049019 and 10.6019/PXD049019.

### Statistical Analysis

Statistical analysis was performed using Prism 6 (GraphPad Software, Inc.) to calculate the mean, SD, and SE mean. *t* test and one-way ANOVA were performed where appropriate to determine significance (GraphPad Prism, RRID:SCR_002798). Biological and technical replicates are indicated throughout the figure captions. All graphical data are reported as mean ± SEM. *, *P* < 0.05; **, *P* < 0.01; ***, *P* < 0.001; and ***, *P* < 0.0001.

### Data Availability Statement

The mass spectrometry proteomics data have been deposited to the ProteomeXchange Consortium via the PRIDE [1] partner repository with the dataset identifier PXD049019 and 10.6019/PXD049019. The datasets that support the findings of this study are available from the corresponding author upon request.

## Results

### Physiologically Relevant Tissue Stiffness Impacts EV Secretion in Patients

**FIGURE 1:**
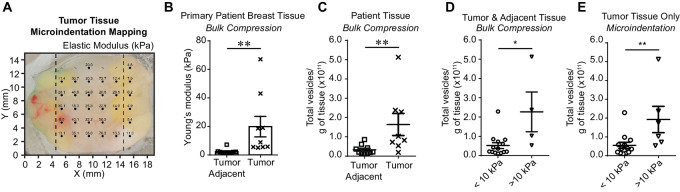
Matrix stiffness impacts the quantity of EVs produced by patient tissue. **A,** Representative primary breast tumor tissue. Sample has been mechanically mapped using microindentation. Dark circles indicate measurements of the elastic modulus expressed in units of kPa. Crosses are nonmeasurable indentations. Dotted lines indicate where the tissue was sectioned into stiff (middle) and soft (right) regions for subsequent vesicle collection. **B,** Mean compression measurements (kPa, mean ± SEM) of patient tumor and adjacent normal tissues. Ten adjacent normal tissue samples and nine tumor tissue samples. Nonparametric *t* test. **C,** Vesicles released per gram of tissue in pathologist determined tumor adjacent or tumor tissue patient samples. Ten normal tissue samples and eight tumor tissue samples. **D,** Combined tumor-adjacent and tumor tissues samples separated by mean compression measurements (kPa, mean ± SEM). Fourteen samples < 10 kPa and four samples > 10 kPa. **E,** Tumor samples separated by mean microindentation measurements (kPa, mean ± SEM). Thirteen samples < 10 kPa and six samples > 10 kPa. Nonparametric *t* test.

To determine physiologically relevant stiffnesses, we obtained primary patient breast tumor and adjacent normal tissues sectioned by a pathologist for mechanical measurements (Fig. 1A). Using a bulk compression test, a method that utilizes uniaxial compression, we found a statistically significant difference in the mean Young modulus of tumor tissues (19.9 ± 7.1 kPa) and tumor adjacent tissues (2.4 ± 0.5 kPa; Fig. 1B). In addition, tumor samples released significantly more vesicles per gram of tissue than tumor adjacent tissue (Fig. 1C; Supplementary Fig. S1A). Tumor tissue stiffness was further mapped using microindentation, a method that determines the local elastic modulus of evenly spaced points (Fig. 1A; Supplementary Fig. S1B). To investigate the effect of tumor stiffness on EVs, we separated the stiff sections (24.4 ± 4.4 kPa, mean ± SEM) and the soft sections (5.7 ± 0.4 kPa) of the tumor tissues based on the microindentation results (Fig. 1D). We noted significant intratumoral and intertumoral heterogeneity, ranging from 2.9 to 76.0 kPa (Supplementary Fig. S1B). On the basis of these findings, we elected to use a 25 kPa matrix to represent stiffer human tumor tissue in our subsequent assays. Given our interest in investigating the impact of EVs at distant sites, such as the lung which has a stiffness ranging from 0.5 to 5 kPa (47, 64–69), we chose a matrix stiffness of 0.5 kPa to represent softer tissues. We compared EVs collected from cells grown on matrices at these physiologic stiffnesses to EVs derived from cells grown on plastic culture dishes with non-physiologic stiffness between 2 and 4 GPa (7).

Following microindentation analysis of resected human breast cancer samples, we sectioned the tissues by stiffness and isolated EVs from stiff and soft regions. To preserve the integrity and micromechanics of these tissues, the tissues were not dissociated; therefore, isolated vesicles were released from both cancer and tumor-associated cells. Significantly more vesicles were released per gram of tissue with a mean tissue stiffness > 10 kPa than from tissues < 10 kPa (Fig. 1D and E; Supplementary Fig. S1A).

### Matrix Stiffness Impacts EV Quantity and Protein Cargo

**FIGURE 2:**
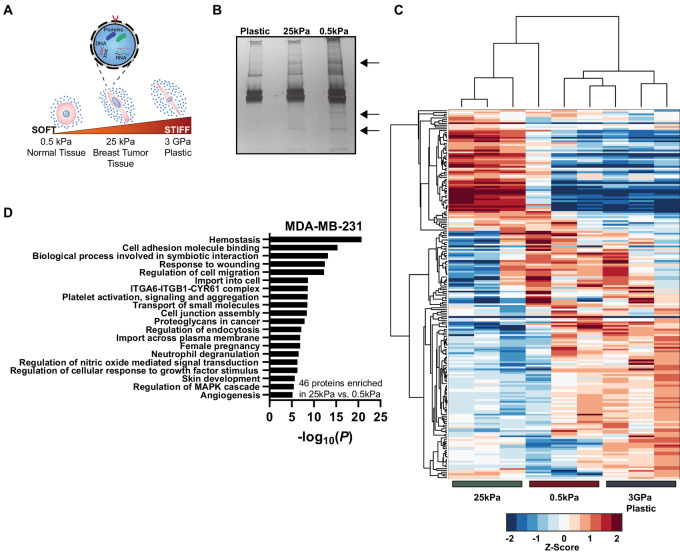
EV cargo is affected by matrix stiffness. **A,** Schematic of EV secretion by cancer cells on standard plastic, tumor (25 kPa) stiffness and normal tissue (0.5 kPa) stiffness. **B,** Silver stain of EV isolated proteins. **C,** Clustergram of EV protein abundance values. The color scale corresponds to the log_2_ normalized z-scored abundance values scaled to −2 and 2 for visual clarity. Three biological repeats. See also Supplementary Table S2. **D,** Gene ontology pathway analysis using Metascape for MDA-MB-231 EV proteins enriched in stiff EVs over soft EVs (79). Three biological repeats.

Above, we determined that tissue stiffness impacts the quantity of vesicles released in breast tumors. Next, we investigated whether matrix stiffness affects EV morphology and protein cargo. Hereafter, we interchangeably refer to EVs released by cells on the plastic matrix as “plastic EVs,” 25 kPa matrix as “stiff EVs,” and 0.5 kPa matrix as “soft EVs.” We compared plastic, stiff, and soft EVs derived from highly metastatic, triple-negative-breast cancer (TNBC) cell lines MDA-MB-231 as our primary model systems (Fig. 2A). We first verified that breast cancer cells displayed the expected stiffness-dependent morphology (18, 70), including a spindle shape on stiffer matrices and a round morphology on the soft matrix, prior to vesicle collection (Supplementary Fig. S2A).

The size of plastic, stiff, and soft EVs was determined using both NTA and transmission electron microscopy (TEM). NTA showed that the mean size of collected particles was between 100 and 150 nm for the TNBC and pancreatic cancer cells across tested matrix stiffnesses (Supplementary Fig. S2B). Corroborating NTA, TEM indicated that plastic, stiff, and soft EVs showed the expected size and morphology of EVs (Supplementary Fig. S2C and S2D; refs. 71, 72). Analysis of the TEM images using a machine learning algorithm (73) confirmed that size and shape of EVs were independent of matrix stiffness (Supplementary Fig. S2D and S2E). Western blots of EV-specific markers (37, 74) confirmed that plastic, stiff, and soft EVs contained tetraspanin cluster of differentiation 63 (CD63) and tumor susceptibility gene 101 (TSG101) across all tested matrix stiffnesses (Supplementary Fig. S2F). While there were similarities in size and morphology, we found major differences in protein content of EVs produced by cancer cells on plastic, stiff, and soft matrices. We determined that there was a nonsignificant difference between the total number of vesicles and the amount of isolated protein between EV conditions (Supplementary Fig. S2G). We then loaded silver-stained electrophoresis gels based on total vesicular protein to normalize for changes in vesicle number and identified qualitative protein cargo differences in the EVs as a function of overall matrix stiffness (Fig. 2B). To test the generality of these findings, we selected pancreatic cancer cell line BxPC3 given that an increase in stiffness is also linked to a poor prognosis in this disease, tumor progression is often characterized by significant changes in the ECM due to desmoplasia, and the selected physiologic stiffnesses also match that of normal tissue and extremely stiff tissue in the pancreas (18, 75–78). We find that the pancreatic cancer cells display the expected morphology on matrices of different stiffness (Supplementary Fig. S2A), the vesicle size and size distribution is independent of matrix stiffness (Supplementary Fig. S2B), the vesicles contain CD63 and TSG101 across all conditions (Supplementary Fig. S2F), and that proteins are differentially enriched in the BxPC3 EVs as a function of overall matrix stiffness (Supplementary Fig. S2H).

To quantify the observed variations in protein content in the breast cancer-derived EVs, we performed mass spectrometry on plastic, stiff, and soft EVs (Fig. 2C; Supplementary Table S2). Proteomic analysis of the EVs identified over 200 proteins expressed in plastic, stiff, and soft EVs. Unsupervised clustering of normalized protein abundances revealed significant variations in content between the three conditions (Fig. 2C; Supplementary Table S2). Using an abundance ratio > 2-fold, we found only two proteins enriched in plastic-derived EVs over stiff EVs, while 52 proteins were enriched in stiff EVs over plastic EVs (Supplementary Fig. S3A). When comparing the physiologically relevant stiffnesses, three proteins were enriched in soft EVs over stiff EVs, and 46 proteins were enriched in stiff EVs over soft EVs (Supplementary Fig. S3B). In the final comparison, six proteins were enriched in plastic EVs relative to soft EVs, and nine enriched in soft EVs relative to plastic EVs (Supplementary Fig. S3C). Gene ontology analysis of proteins enriched in stiff EVs identified pathways related to the immune response, tumorigenesis, adhesion, and metastasis including response to wounding, ECM–receptor interaction, cell-junction organization, integrin complexes, and cellular response to IFNγ and IL12 (Fig. 2D; Supplementary Fig. S3D; ref. 79).

The vesicle protein content indicates that plastic, stiff, and soft EVs may have different functional roles in promoting metastasis. Given that many of the pathways enhanced in the stiff over soft EVs overlapped with cell adhesion and cell–ECM interactions (Fig. 2D), we hypothesized that these matrix stiffness–dependent variations could impact EV biodistribution to different organs (lung, liver, etc.) and the ensuing spread of cancer cells from the primary tumor to these organs. Furthermore, we decided to focus herein on the stiff and soft EVs as the plastic EVs are less physiologically relevant, yielding different cargo that could impact functional assays.

### Stiff EVs Show Enhanced Biodistribution *In Vivo*

**FIGURE 3:**
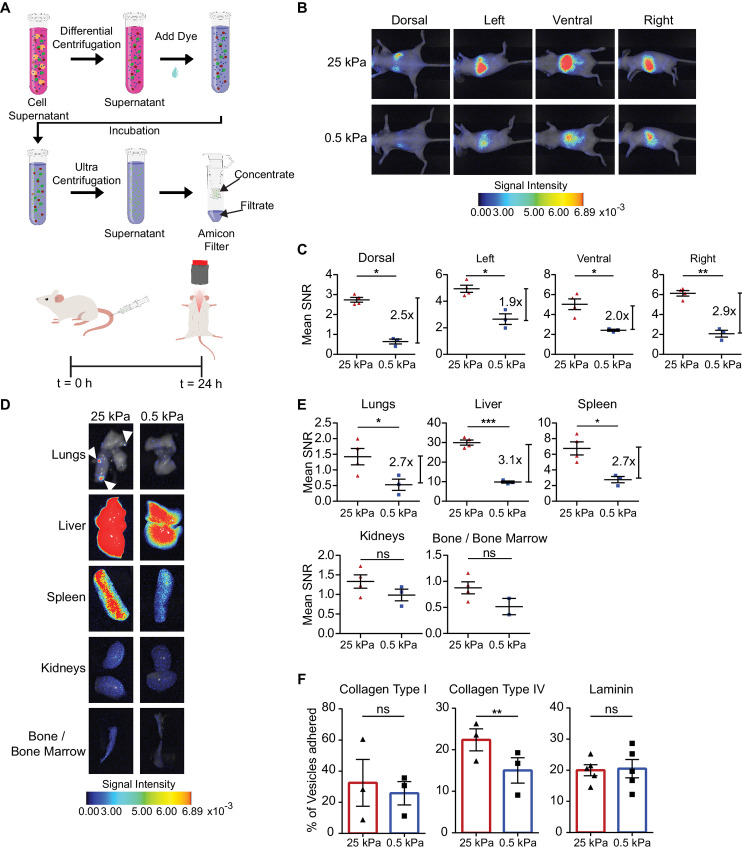
Stiff EVs show increased biodistribution and retention *in vivo.***A,** Schematic of EV isolation and staining prior to tail-vein injection of 10 µg of EVs in nude mice. NIR imaging (**B**) and mean SNR (**C**) of MDA-MB-231 vesicle biodistribution in dorsal, left, ventral, and right sides (mean ± SEM). Signal intensity is in arbitrary units (a.u.). Three mice in 0.5 kPa condition, and four in 25 kPa condition; one-way ANOVA. NIR imaging (**D**) and mean SNR biodistribution (**E**) in the lungs, liver, spleen, kidneys, and bone/bone marrow (mean ± SEM). Signal intensity is in arbitrary units (a.u.). Three mice in MDA-MB-231 0.5 kPa condition and four in 25 kPa condition; one-way ANOVA. **F,** MDA-MB-231 EV binding assay to ECM proteins collagen type I, collagen type IV, and laminin (mean ± SEM). Three biological repeats for collagen type I and collagen type IV; five for laminin. Paired *t* test.

To determine whether overall differences in molecular cargo, prompted by differing matrix stiffness, had a functional effect on the distribution and retention of breast cancer–derived EVs *in vivo*, we injected immunodeficient nude mice with fluorescent EVs via their tail veins (Fig. 3A). We chose a tail vein metastasis model, which forces metastasis to the lung, given our focus on near-infrared (NIR) from the dorsal, left, ventral, and right sides (Fig. 3B; Supplementary Fig. S4A). For all angles, the mean SNR was 2- to 3-fold greater for stiff EVs compared with soft EVs (Fig. 3C). In addition to lungs, we observed a 3-fold increase in the mean SNR for stiff EVs over soft EVs in primary filter organs—liver and spleen (Fig. 3D and E). Non-physiologic plastic EVs shared a similar biodistribution profile to 25 kPa (Supplementary Fig. S4A and S4B), with decreased retention in the liver (Supplementary Fig. S4C and S4D).

To identify the mechanism driving this stiffness-mediated EV biodistribution, we investigated whether the stiff and soft EVs bound differentially to ECM proteins, especially ECM molecules associated with tumor progression and metastasis (80, 81). Via quantification of total fluorescent signal, stiff EVs preferentially bound to collagen type IV relative to soft EVs (Fig. 3F). The stiff and soft EVs did not demonstrate significant differences in binding to collagen type I or laminin (Fig. 3F). On the basis of the enrichment of adhesion molecules in stiff EVs in the proteomics data, ITGα_2_β_1_ and CD44 could facilitate the enhanced binding to collagen type IV (81–86, 87).

### Stiff EVs Promote Cancer Cell Dissemination and Survival *In Vivo*

Because breast cancer–derived stiff EVs were retained within common secondary sites to a much greater extent than soft EVs, and the stiff EVs bound preferentially to ECM proteins linked to metastasis, we sought to determine whether the EVs would directly affect cancer cell behavior during metastasis. To explore this scenario, we created a zebrafish xenograft model to explore cancer cell dissemination and survival *in vivo*. Zebrafish possess orthologs for 70% of human genes, are translucent allowing for real-time *in vivo* visualization, cost-effective, and lack adaptive immune systems during early embryogenesis, highlighting their utility as effective xenograft hosts. We utilize zebrafish embryos 2 days postfertilization (2 dpf).

**FIGURE 4:**
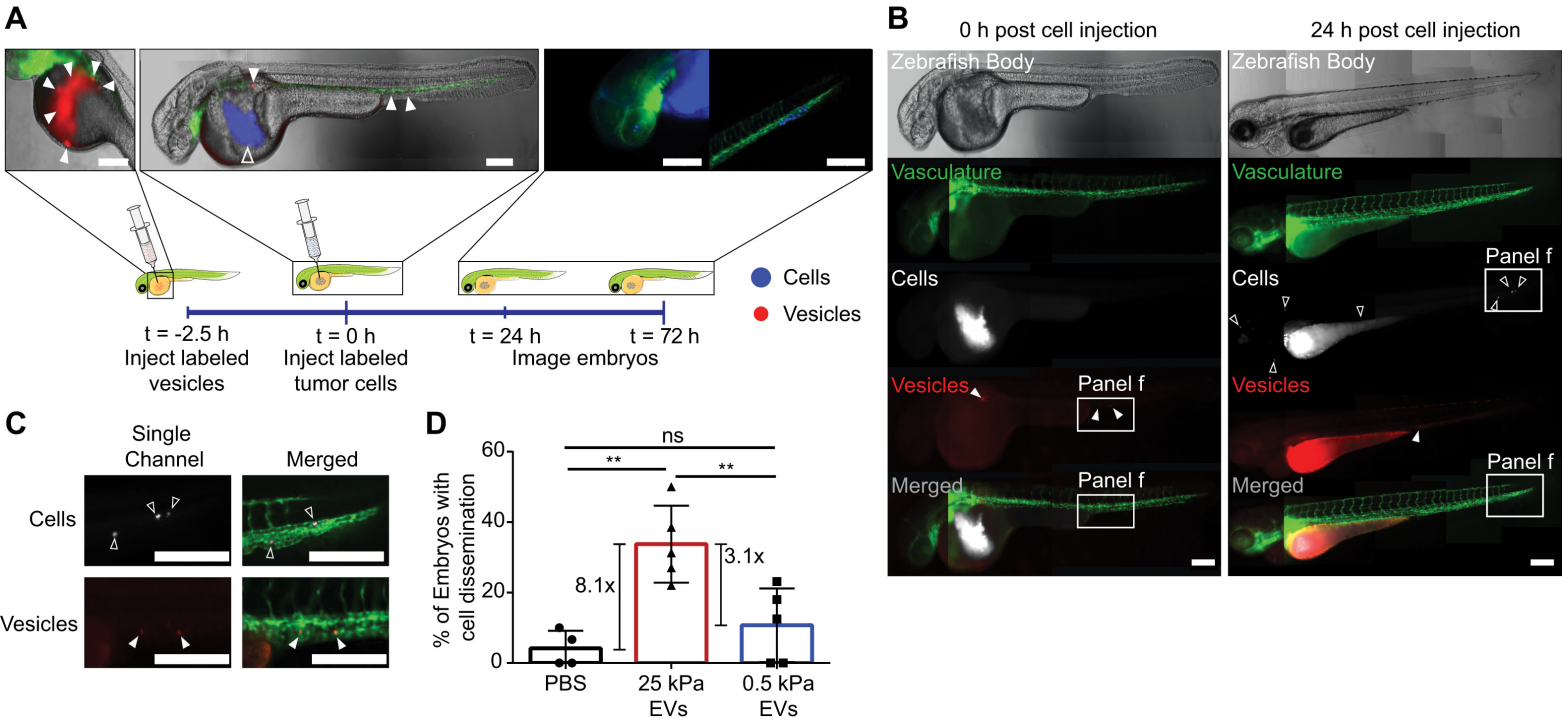
Stiff EVs promote cancer cell migration and dissemination. **A,** Schematic of 2-day GFP+ zebrafish embryo model system used to test the ability of MDA-MB-231 EVs (red, filled triangles) to disperse MDA-MB-231 cancer cells (blue, open triangles). Scale bar, 100 µm. **B,** Full zebrafish body images of the embryos at time 0 and 24 hours postinjection of MDA-MB-231 cancer cells. Scale bar, 100 µm. Representative images. **C,** Enlarged images of disseminated MDA-MB-231 cells (white) and EVs (red). Scale bar, 100 µm. Representative images. **D,** Percentage of injected embryos with cancer cell dissemination to the head or the tail. Total number of fish per condition is 47 for PBS control, 63 for 25 kPa, and 78 for 0.5 kPa condition. Five biological repeats of EVs. One-way ANOVA.

PBS, breast cancer–derived stiff EVs, or breast cancer–derived soft EVs were injected into the yolk sac of zebrafish embryos 2 dpf, followed by injection of cancer cells within 2–2.5 hours of EV injection (Fig. 4A). Embryos were imaged at 24 hours to quantify cell dissemination (Fig. 4B and C). Quantification of embryos displaying cancer cell dissemination to the head and tail 24 hours after cell injection in a dye-only condition (no preinjected vesicles) showed that only 4.2% of fish exhibited a net transfer of cells out of the yolk sac to the head or tail of the fish (Fig. 4D). In contrast, 33.8% and 10.7% of fish preinjected with stiff and soft EVs, respectively, had disseminated cancer cells (Fig. 4D). Fish injected with plastic EVs had negligible dissemination (Supplementary Fig. S4E). These results suggest there is a role for EVs in mediating metastasis which depends critically on the physical properties of the microenvironment.

### Soft EVs Transform Fibroblasts Into CAF-like Cells

**FIGURE 5:**
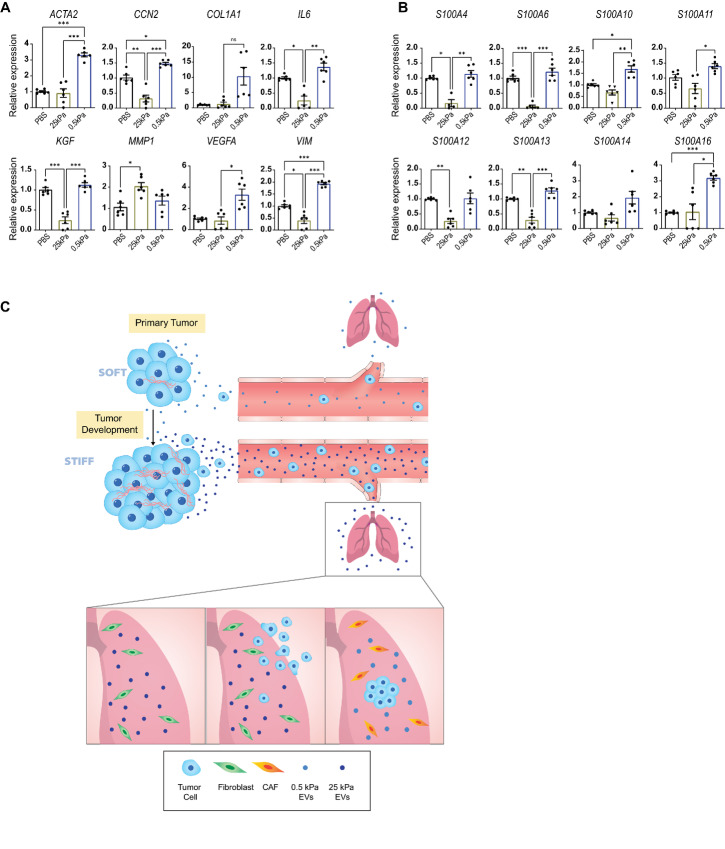
Soft EVs transform the phenotype of resident lung fibroblasts. **A** and **B,** Gene expression fold change in *ACTA2*, *CCN2*, *COL1A1*, *IL6*, *KGF*, *MMP1*, *VIM*, *VEGFA*, *S100A4, S100A6, S100A10, S100A11, S100A12, S100A13, S100A14, S100A16* assessed by qRT-PCR in IMR90 human lung fibroblasts exposed to PBS only, 25 kPa EVs, and 0.5 kPa EVs. Gene expression data normalized to PBS condition. Two biological repeats. Welch ANOVA. **C,** Schematic showing the arrival of stiff EVs in the lung after being secreted from the primary tumor. Left panel shows a mechanically soft environment, and EVs encountering resident normal lung fibroblasts; (middle) cancer cells are then recruited to the lung; and (right) the cells, now experiencing a soft environment, release soft EVs that transform the resident fibroblasts to a CAF phenotype.

Because breast cancer–derived stiff EVs facilitate the dissemination of cancer cells (Fig. 4), we wanted to assess whether stiff and soft EVs would differentially affect the ability of cancer cells to form tumors at secondary sites by transforming resident stromal cells, particularly fibroblasts. Fibroblasts are responsible for maintaining homeostasis as immunoregulatory cells and through the generation of structural ECM molecules like collagen I (88–90). Because we observed the greatest differences in EV retention in the lungs, liver, and spleen, (Fig. 3E) and breast cancer frequently metastasizes to the lung *in vivo*, we assessed EV-mediated changes in the phenotype of normal lung fibroblasts (Fig. 5A and B). Cancer cells recruited to the lungs are exposed to a relatively soft microenvironment (0.5–1 kPa) at this distant site, which has a stiffness like that of normal breast tissues (91, 92).

To determine how the mechanically new environment of the lung may promote further tumor progression, we investigated how stiff and soft EVs differentially modulated resident lung fibroblasts by assessing the expression of a several CAF markers (27, 88–90, 93–97). Lung fibroblasts treated with stiff and soft EVs showed significantly different abilities to induce CAF-associated markers. Soft EVs significantly upregulated CAF-associated molecule (27, 88, 93–96) smooth muscle actin (*ACTA2*) 3.3-fold, connective tissue growth factor (*CCN2*) 1.5-fold, and vimentin (*VIM*) 2-fold relative to a vesicle-free condition (Fig. 5A). Interestingly, only matrix metalloproteinase 1 (*MMP1)* was upregulated by Stiff EVs (Fig. 5A). Instead, stiff EVs downregulated many of these CAF-associated genes including: *CCN2, IL6, KGF,* and *VIM* (Fig. 5A). While there was an approximately 10-fold increase in collagen type I expression from soft EVs, the difference was not significant (Fig. 5A). Comparatively, soft EVs had significantly higher ability to modulate *ACTA2, CCN2, IL6, KGF, VEGFA,* and *VIM* relative to stiff EVs.

We then probed how stiff and soft EVs regulated normal lung fibroblast gene expression of S100 proteins. In breast and pancreatic cancers, dysregulation of S100 protein expression, due in part to CAFs, is tied to an increase in growth, metastasis, and angiogenesis (98). Previously, the success of premetastatic niche formation in the lung was determined to be dependent on S100 protein upregulation (99). In lung fibroblasts exposed to soft EVs, we observed a noticeable upregulation of two S100 genes (*S100A10* and *S100A16*; Fig. 5B) compared with a vesicle-free condition. Our results also indicate that stiff EVs downregulate the expression of *S100A4*, *S100A6, S100A12*, and *S100A13.* When compared with 0.5 kPa EVs, we observed a 6.8-fold (*S100A4)*, 19.2-fold (*S100A6)*, 3.8-fold (*S100A12)*, and 4.3-fold (*S100A13*) decrease in expression (Fig. 5B). Together, these results suggest that soft vesicles produced by newly disseminated cancer cells in the soft microenvironment of the lung are significantly more effective at producing a CAF-like phenotype in lung fibroblasts via ensuing upregulation of S100 inflammatory signals and *ACTA2*//*VEGFA/VIM* (Fig. 5C).

## Discussion

This work demonstrates the importance of utilizing physiologically relevant conditions for studying the role of EVs in cancer. EVs released by cancer cells on plastic dishes differentially expressed hundreds of proteins, resulting in inaccurate information about the ability of vesicles to distribute *in vivo* and promote cell dissemination compared with matrices that mimic tissue stiffness at the primary and distant sites (Figs. 3 and 4). Our stiff tumor tissue and soft normal tissue matrices significantly altered EV quantity, protein cargo, function, and their potential to affect multiple aspects of the metastatic cascade.

The quantification of vesicles from primary patient breast tissue indicates that more EVs are released in stiff tissue over soft tissue. Within the tissue, there are many different cell types, all contributing to the number of small EVs we isolated in this study. As of now, there are no effective methods or markers for separating EVs based on the cell type of origin, which limits our ability to determine the number of vesicles produced by each cell type of the tumor microenvironment. We do notice though that there is an increase in cell number and density within the stiff breast tumor tissue compared with the soft breast tumor tissue (61).This result suggests increased cancer cell density may contribute to the observed EV secretion. Recent work done in hepatocellular carcinoma suggests this increased EV secretion on stiff environments is driven by activation of the Akt signaling pathway contributing to Rab8 mediated EV secretion (53).

In addition to the observed variations in stiffness-dependent vesicle secretion in breast cancer, the protein cargo of EVs critically depends on matrix stiffness. We identified a 3-fold increase in mean SNR between the stiff and soft EVs in the lungs and liver, two of the most common sites of breast cancer metastasis. These results suggest that small EVs have a different rate of clearance *in vivo* as a function of stiffness, presumably due to our observed stiffness-dependent presentation of adhesion molecules on EVs and their ECM binding affinity (Fig 3F; Supplementary Fig. S2A). The increased retention of stiff EVs in the lung could be a function of specific integrins, including α_6_β_4_ and α_6_β_1_, which have been previously linked to organotropic homing (48). Collagen type I, collagen type IV, and laminin have all been linked previously to cancer cell migration and invasion (80–82, 100–107). Collagen type IV lines all basement membranes in the liver and airway basement membrane in the lung (108, 109). Preferential binding of stiff EVs to basement-membrane proteins may, therefore, also explain increased stiff EV retention in the lungs and liver. Previously, small EVs were shown to increase lung vascular permeability *in vivo* (48). Our results suggest that stiff EVs, which bind these basement-membrane proteins promote cancer dissemination into the lung. This EV-mediated metastasis may be driven by thrombospondin-1 (*THBS1*) which overexpressed in stiff EVs (Supplementary Fig. S3B and S3C) was observed to regulate cancer cell motility (54). However, further studies are needed to delineate the mechanism driving soft EV retention in distal sites.

On the basis of zebrafish experiments, the mechanism driving EV-mediated cell movement is matrix dependent. Compared with soft EVs, stiff EVs demonstrated an enhanced ability to induce cancer dissemination *in vivo* (Fig. 4A–D). These findings in unison with EV proteomic data suggest that the proteins responsible for cell spreading from the primary tumor are preferentially sorted into EVs released by cancer cells experiencing a stiff tissue matrix.

While stiff EVs are more effective at promoting early step of the metastatic cascade through dissemination, we determined that stiff and soft EVs operate in a dynamic way to colonize distant organs, especially the lung. Once internalized by normal lung fibroblasts, the stiff EVs downregulate S100 expression, while soft EVs upregulate activation, vasculogenic, and inflammatory markers in the fibroblasts. The increased retention of stiff EVs in the lung and the downregulation of S100 proteins in normal resident lung fibroblasts may seem counterintuitive; however, a decrease in S100A4 expression has been linked to blocking fibroblast invasion and T-cell recruitment at the primary tumor (110). In addition, there is a direct relationship between the expressions of VEGFA and S100A4 in fibroblasts, with the expression of both molecules being important for metastatic colonization (111). Decreased *S100A6* expression in breast cancer has been linked to a worse prognosis regardless of subtype (112). Although little has been studied about its role in breast cancer (112), S100A13 is known to regulate fibroblast growth factor (FGF1) and IL1α, which can affect the angiogenic and mitogenic properties of the tumor microenvironment (113–115). Therefore, the decrease in the expression of these S100 proteins in fibroblasts can promote a prometastatic environment in the lung, prior to the arrival of cancer cells. However, any functional changes observed in EV-modulated CAFs remain to be explored.

Once cancer cells arrive in the new soft environment of the lung, they release soft EVs that transform the resident fibroblasts toward a CAF phenotype through increased expression of *ACTA2*, *COL1A1*, and *VEGFA, VIM* (Fig. 5A). This interaction between soft environment and fibroblasts could also take place during early tumor progression or at other distant sites of metastasis (47, 64–69). The soft EVs demonstrate an upregulation of cytoskeletal regulating, binding, and cell signaling proteins linked to primary tumor growth (Fig. 5A and B). We propose that stiff EVs direct the recruitment of cancer cells and ensure retention in the lung by generating an anti-inflammatory environment; once there, the cancer cells experience a soft matrix and release soft EVs that transform the surrounding stroma to a protumorigenic environment. However, currently we are unable to delineate which cell types, if any, preferentially take up stiff and soft EVs. Further internalization studies are needed to understand EV cell type interactions.

Together our results indicate that EVs promote metastasis through multiple mechanisms that take advantage of the differences in stiffness of the primary and metastatic sites. The first is through the increased retention and biodistribution of stiff EVs *in vivo*, due to augmented binding to the ECM via increased integrin presentation, which allows for the formation of premetastatic niches. Second, EVs produced at both normal and tumor tissue stiffnesses affect changes to the surrounding ECM by regulating fibroblast activity. Stiff EVs decrease inflammatory signaling in the fibroblasts to facilitate cancer cell arrival at the lung, and once inside the mechanically soft lung, cancer cells release soft EVs that increase the expression of protumorigenic markers in the fibroblasts. Our findings highlight the critical importance of the physical properties of the ECM on the quantity, quality, and function of EVs produced by cancer cells in mediating their metastasis. Future exploration may focus on investigating pan-cancer markers of EV-driven stiffness-mediated metastasis for diagnostic and therapeutic applications.

## Supplementary Material

Supplementary Methods Additional Methods

Table S1 Table S1 - Primers for qRT-PCR analysis

Table S2 Master protein list of identified proteins present in TMT analysis.

Figure S1 Stiffness measurements of primary patient tissues

Figure S2 Isolation and characterization of EVs

Figure S3 Comparison of protein cargo for plastic, stiff and soft EVs

Figure S4 Plastic EVs have different biodistribution compared to stiff and soft EVs.
