# Supplementary material for: Small Extracellular Vesicles Promote Stiffness-mediated Metastasis
Source: Cancer Res Commun. 2024 May 9;4(5):1240–52. doi: 10.1158/2767-9764.CRC-23-0431 (PMC11080964; doi:10.1158/2767-9764.CRC-23-0431)
Supplement: Supplementary Methods — Additional Methods [file crc-23-0431-s01.pdf]

Supplementary Methods

Size distributions of EVs

Samples were initially diluted at 1:20 with DPBS to achieve a concentration below 10^9 particles/ml. Samples were introduced in the instrument using a syringe attached to a pump. For each sample, three videos were captured for 60 s with a camera level between 13 and 16. All measurements were carried out at room temperature and the chamber was cleaned with 10% ethanol and DI water between each sample. Videos were analyzed using NanoSight with detection threshold set within 5 to 10 to obtain vesicle concentration (particles/ml) and size distribution (nm).

Tumor stiffness mapping using microindentation

The tumor section was mounted on a customized stage and DPBS was applied to keep the tissue hydrated throughout the measurement. Dynamic indentation by nanoindenter (Nanomechanics Inc.) was used to characterize the tumor elastic modulus (Akhtar *et al.*, 2018). Sneddon's stiffness equation (Sneddon, 1965) was applied to relate dynamic stiffness of the contact to the elastic storage modulus of the samples (Herbert, Oliver and Pharr, 2008; Herbert *et al.*, 2009). 500 µm flat cylindrical probe was used in the indentation experiments. Briefly, procedure of indentation is comprised of 3 steps: 1) approaching and finding tissue surface at the indenter's resonant frequency to enhance contact sensitivity and accuracy, 2) pre-compression of 50 µm to ensure good contact, 3) dynamic measurement at 100 Hz oscillation frequency with amplitude of 250 nm. The indentation procedure mentioned above was done consecutively on multiple regions of a single tissue surface in a grid pattern to obtain stiffness map of the tumor. Because obtaining a perfectly flat tissue surface was difficult due to tissue heterogeneity, individual indentation processes were observed using a microscope camera to determine inappropriate contact of the probe to the tissue for inaccurate measurement which were excluded from data. Typically, the number of indentation points per tissue mapping was 20-40 with the resolution of 1-2 mm spacing between points depending on the size of tumor sample. The duration of stiffness mapping was 30 min on average.

Patient tissue vesicle collection and characterization

After collection in DPBS, primary patient tissue samples underwent bulk compression and microindentation to determine stiffness. Each sample was then transferred to 5 mL of 1% penicillin-streptomycin solution in 013-CV DMEM and incubated at 37ºC overnight. After 24 h, the supernatant of each sample was collected and the patient tissue was transferred to 120 mL of formalin and left at room temperature. The supernatants then underwent differential centrifugation to pellet out cells and cellular debris, being spun down at the following settings: 4ºC at 800 g for 5 min, 2,000 g for 10 min and 10,000 g for 30 min. The supernatants were isolated after every spin and at the end of the 10,000 g spin, they were filtered using 0.22 µm PES filters (Genesee) into an Amicon ^{R} Ultra - 15 10 K centrifugal filter. The Amicon spin was conducted at the setting of 4ºC at 5,000 g for 40 min, per manufacturer protocol. After the final spin, the concentrates were measured and deposited into eppendorfs. 50 µl of each sample was placed in a separate eppendorf with 950 µl of PBS, to create a sample with a dilution factor of 20. The diluted samples were then taken for nanoparticle tracking analysis and diluted further as needed.

EV proteomics

EVs were collected from three biological replicates of cell cultures grown on tissue culture plastic, 25 kPa, and 0.5 kPa matrices. Bligh-Dyer extraction was used to precipitate the protein content, while lipids and polar metabolites were removed in the organic (lower) and aqueous (upper phases) (Bligh, E.G. and Dyer, 1959). Briefly, 200 µL of methanol and 100 µL of chloroform were added, and the samples were vortexed for 1 min and sonicated for 5 min to break the EV structures. 100 µL of chloroform were added followed by 100 µL of water to precipitate the protein contents into the interphase. The samples were centrifuged at 14,000 x g for 10 min at room temperature. After refrigeration at -20ºC (this did not freeze either phase) overnight the precipitated proteins settled in the interphase. The methanol/water (upper) phase, and the lipid-containing chloroform (lower) phase were discarded. The remaining protein containing pellet was lyophilized to dryness and reconstituted in 20 µL of 50 mM triethyl ammonium bicarbonate pH 8.0 in 50% aqueous 2,2,2-triflouroethanol (TFE) to solubilize the precipitated proteins. The samples were reduced in 5 mM TCEP at 37ºC for 30 min and alkylated in 10 mM IAA at room temperature for 15 min in the dark. The sample was diluted 10 x in 50 mM triethyl ammonium bicarbonate

(pH 7.5) and digested with 2 µg of Trypsin / Lys-C Mix at 40ºC for 16 h. Each sample was labeled with 41 µl of a different 10-plex TMT reagent for 90 min. The labeling reactions were quenched with 8 µl 5% hydroxylamine for 15 minutes, and the labeled samples were combined and lyophilized. The pooled sample was reconstituted in 2% acetonitrile, 0.1% trifluoroacetic acid and fractionated by high pH solid phase extraction on an Oasis HLB plate (Waters Corp). The peptides were eluted sequentially off the Oasis plate into separate fractions using solvents containing 5%, 10%, 25%, and 75% acetonitrile in 10 mM triethyl ammonium bicarbonate pH 8.5. The fractions were lyophilized and reconstituted in 50 µL of 0.1% formic acid 2% acetonitrile prior to duplicate LC-MS analysis.

Five microliters (10%) of each fraction were separated over a binary reversed phase gradient using aqueous 2% acetonitrile 0.1% formic acid (mobile phase A), and 0.1% formic acid in 90% acetonitrile (mobile phase B) as follows: Sample loading in 0% B with a rapid jump to 8% B, a linear ramp from 8% to 30% B over 90 min, linear ramp to 48% B over 15 min, ramp to 95% B over 5 min, 9 min hold at 95% B, and drop back to 0% B in 1 min. Data dependent acquisition DDA was used to quantify peptides and consequently proteins. The precursor scan spanning 400-1600 m/z was acquired at 120000 (at m/z = 200) resolution with automatic gain control (AGC) set to 200000 and 50 ms maximum ion injection time (IT). Precursor ions in the +2 to +6 charge states were individually isolated in 0.4 Da isolation windows and serially fragmented in order of highest intensity by high energy collisional dissociation (HCD, 38 normalized collision energy) for 4 s after each precursor scan. Fragment ions were detected from 120-2000 m/z scan at 50000 resolutions, using 50000 AGC and 86 ms maximum IT. Previously fragmented ions were excluded for 30 s to prevent redundant precursor sampling.

The collected data (4 fractions analyzed in duplicate) were combined and searched against the SwissProt *Homo Sapiens* database with Mascot (v.2.6.2 Matrix Science) in Proteome Discoverer 2.4 (RRID:SCR_014477) using 5 ppm precursor and 0.01 Da fragment mass error tolerances, trypsin as enzyme, allowing for two missed cleavages, with cysteine carbamidomethylation and TMT on N-terminal amino acids as fixed modifications, and TMT labeling of lysine, methionine oxidation, and deamination of asparagine and glutamine as variable

modifications. Mascot ".dat" files were validated with Percolator with peptide identifications filtered at <5% FDR. The reporter ions from the Peptide Spectral Matches (PSMs) assigned to proteins were used to calculate, normalize and scale protein abundances across all samples. Relative ratios were calculated from normalized median protein abundances of biological replicates.

The clustergram was created in MATLAB (RRID:SCR_001622) using functions included in the built-in bioinformatics toolbox. Clustered using spearman distances. The heatmap contains all master proteins of high confidence identified in the 9-plex TMT experiment. The color scale corresponds to the log2 normalized z-scored abundance values scaled to -2 and 2 for clarity. Volcano scatter plots were generated in MATLAB. The relative abundance ratio (fold change) represents the median ratio for each master protein across all three biological replicates. Enriched proteins showing a 2-fold or greater change in the numerator are plotted in red, those enriched 2-fold or greater in the denominator are plotted in green. To perform gene ontology, express analysis was run on those proteins enriched 2-fold or greater in Metascape.[69]
