# Supplementary material for: Small Extracellular Vesicles Promote Stiffness-mediated Metastasis
Source: Cancer Res Commun. 2024 May 9;4(5):1240–52. doi: 10.1158/2767-9764.CRC-23-0431 (PMC11080964; doi:10.1158/2767-9764.CRC-23-0431)
Supplement: Table S1 — Primers for qRT-PCR analysis [file crc-23-0431-s02.pdf]

Table S1: Primers for qRT-PCR analysis

Gene	Forward Sequence (5'-3')	Reverse Sequence (5'-3')
ACTA2	GTGTTGCCCCTGAAGAGCAT	GCTGGGACATTGAAAGTCTCA
CCN2	TGGAGTTCAAGTGCCCTGAC	CTCCCACTGCTCCTAAAGCC
COL1A1	TGCTCGTGGAAATGATGGTG	CCTCGCTTTCCTTCCTCTCC
GAPDH	GCACCGTCAAGGCTGAGAAC	GCCTTCTCCATGGTGGTGAA
IL6	ACTCACCTCTTCAGAACGAATTG	CCATCTTTGGAAGGTTCAGGTTG
KGF	AGGCAGACAACAGACATGGAAT	TCGATCCTCAGGTACCACTGT
MMP1	GGGGCTTTGATGTACCCTAGC	TGTCACACGCTTTTGGGGTTT
S100A10	GGGCTTCCAGAGCTTCTTTT	CTTCTATGGGGGAAGCTGTG
S100A11	TGTCCTTGACCGCATGATGAA	TTCTGGGAAGGGACAGCCTT
S100A12	CTTCCACCAATACTCAGTTCGG	GCAATGGCTACCAGGGATATG
S100A13	TTCTTCACCTTTGCAAGGCA	GAGAGCCCACATCCTTGAGC
S100A14	CTCATGCCGAGCAACTGTG	GGGTACAGGGTGGTGGTAGA
S100A16	ATGCTGTCGGACACAGGG	TGATGCCGCCTATCAAGGTC
S100A4	TCTTGGTTTGATCCTGACTGCT	AAGCACGTGTCTGAAGGAGC
S100A6	AAGCTGCAGGATGCTGAAAT	CCCTTGAGGGCTTCATTGTA
TBP	GAGCCAAGAGTGAAGAACAGTC	GCTCCCCACCATATTCTGAATCT
TUBA3C	AGGAGTCCAGATCGGCAATG	GTCCCCACCACCAATGGTTT
VEGFA	AGGGCAGAATCATCACGAAGT	AGGGTCTCGATTGGATGGCA
VIM	AGTCCACTGAGTACCGGAGAC	CATTTCACGCATCTGGCGTTC
