# Supplementary material for: Small Extracellular Vesicles Promote Stiffness-mediated Metastasis
Source: Cancer Res Commun. 2024 May 9;4(5):1240–52. doi: 10.1158/2767-9764.CRC-23-0431 (PMC11080964; doi:10.1158/2767-9764.CRC-23-0431)
Supplement: Table S2 — Master protein list of identified proteins present in TMT analysis. [file crc-23-0431-s03.pdf]

Table S2: Figure 1C master protein list of identified proteins present in all samples: 9-plex TMT analysis.

Protein #	Symbol	#	Symbol	#	Symbol	#	Symbol	#	Symbol
1	C6	46	IGFBP7	91	DSP	136	LYZ	181	KRT17
2	CASP14	47	S100A7	92	FGB	137	CP	182	TXN
3	HBA1	48	CFAP100	93	CD9	138	CALML5	183	CD109
4	LAMP2	49	PTX3	94	PGK1	139	CCN1	184	BSG
5	ITIH2	50	SERPINA10	95	XPO1	140	PPIA	185	FEN1
6	POSTN	51	SERPIND1	96	AHNAK	141	RHOC	186	QSOX1
7	SERPINF2	52	HS6ST1	97	AZGP1	142	C7	187	TUBB
8	HSPG2	53	ITGA3	98	CPA6	143	MFGE8	188	APOH
9	HSP90AA1	54	HGFAC	99	TUBA1A	144	HSPA1B	189	PCLO
10	DMKN	55	S100A8	100	HSPA8	145	AMY2A	190	GC
11	EDIL3	56	KRT1	101	VNN1	146	H1-4	191	H2BC17
12	HSP90B1	57	C3	102	GSN	147	H1-2	192	NCAM1
13	HLA-B	58	UBA52	103	S100A9	148	KNG1	193	APOB
14	TGM3	59	KRT6B	104	CDC42	149	FLG	194	ITIH4
15	MCAM	60	KRT6A	105	ANXA2	150	ACTA1	195	C4B
16	SUPT6H	61	LUM	106	EEF1A1	151	F3	196	MT1G
17	FBLN1	62	SLC43A3	107	CD59	152	GPI	197	KRT78
18	AHSG	63	SOD1	108	ADAMTS13	153	KIF2B	198	CD82
19	TGM1	64	EGFR	109	ACTB	154	SLC39A10	199	FABP5
20	THBS1	65	THBS4	110	LOC102723996	155	SBSN	200	KRT4
21	SERPINA7	66	ITGB4	111	EFEMP1	156	EEF2	201	AFP
22	CD44	67	HMGN2	112	KRT13	157	NT5E	202	SLC2A14
23	PZP	68	NPTN	113	TF	158	LGALS7B	203	TPM3
24	PLTP	69	PPARD	114	FSTL1	159	H2AX	204	ITGB1
25	LGALS1	70	HBB	115	H2BC5	160	PRDX1	205	KRT9
26	AHSG	71	MYADM	116	LMNA	161	CD47	206	KRT16
27	ALB	72	TMOD2	117	MRC2	162	TMSB10	207	PRDX2
28	SFN	73	F13A1	118	CD63	163	CHIA	208	MARCKS
29	CD81	74	KRT80	119	GAPDH	164	DHRS4	209	IL1RAP
30	VTN	75	BMP1	120	BASP1	165	ART4	210	DSC1
31	SLC16A3	76	KRT14	121	ENO1	166	APMAP	211	GNB2
32	SLC1A5	77	RBP4	122	PGM1	167	A2M	212	RGN
33	H2AZ1	78	ATP1A1	123	SLC3A2	168	SHBG	213	CTSD
34	LSR	79	FLG2	124	MSN	169	CNTN1	214	HNRNPA1
35	F10	80	COL5A1	125	COL1A1	170	H3C13	215	C9
36	KRT2	81	DSG1	126	PLG	171	HABP2	216	COL6A1
37	BRD9	82	CLEC3B	127	C5	172	DCD	217	AFM
38	CLSTN1	83	A1BG	128	MT1B	173	FAP	218	KRT5
39	ATP7B	84	VNN2	129	F2	174	SLC2A1	219	LGALS3BP
40	AGRN	85	ATP2B4	130	PFN1	175	ALDOB	220	ITGA2
41	TIMP1	86	GNB1	131	COMP	176	MAGEC3	221	TUBA4A
42	FLNB	87	SERPINC1	132	H4C15	177	SERPINF1	222	IGF2R
43	HRNR	88	PODXL	133	CAPN2	178	LTF	223	SERPINB12
44	CDH13	89	TRAP1	134	CSTA	179	ITIH1	224	TOP1
45	ITGA6	90	LHFPL2	135	ARHGAP35	180	TIMP2		
