# Supplementary material for: Small Extracellular Vesicles Promote Stiffness-mediated Metastasis
Source: Cancer Res Commun. 2024 May 9;4(5):1240–52. doi: 10.1158/2767-9764.CRC-23-0431 (PMC11080964; doi:10.1158/2767-9764.CRC-23-0431)
Supplement: Figure S1 — Stiffness measurements of primary patient tissues [file crc-23-0431-s04.pdf]

Figure S1: Stiffness measurements of primary patient tumor tissues

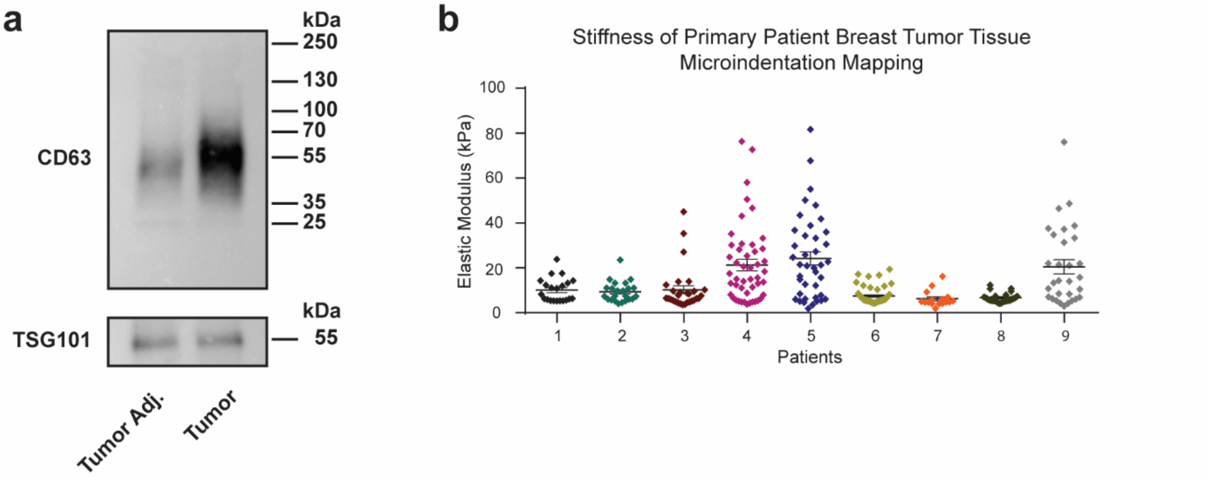

Figure S1: Stiffness measurements of primary patient tumor tissues.

(a), Representative western blots of EV markers CD63 and TSG101 for EVs isolated from patient tumor adjacent and tumor tissue. (**b**), Microindentation measurements (kPa, mean ± SEM) for each breast-cancer patient tissue tumor sample. Nine patients.
