# Supplementary material for: Small Extracellular Vesicles Promote Stiffness-mediated Metastasis
Source: Cancer Res Commun. 2024 May 9;4(5):1240–52. doi: 10.1158/2767-9764.CRC-23-0431 (PMC11080964; doi:10.1158/2767-9764.CRC-23-0431)
Supplement: Figure S2 — Isolation and characterization of EVs [file crc-23-0431-s05.pdf]

Figure S2: Isolation and characterization of EVs produced by cancer cells *in vitro*

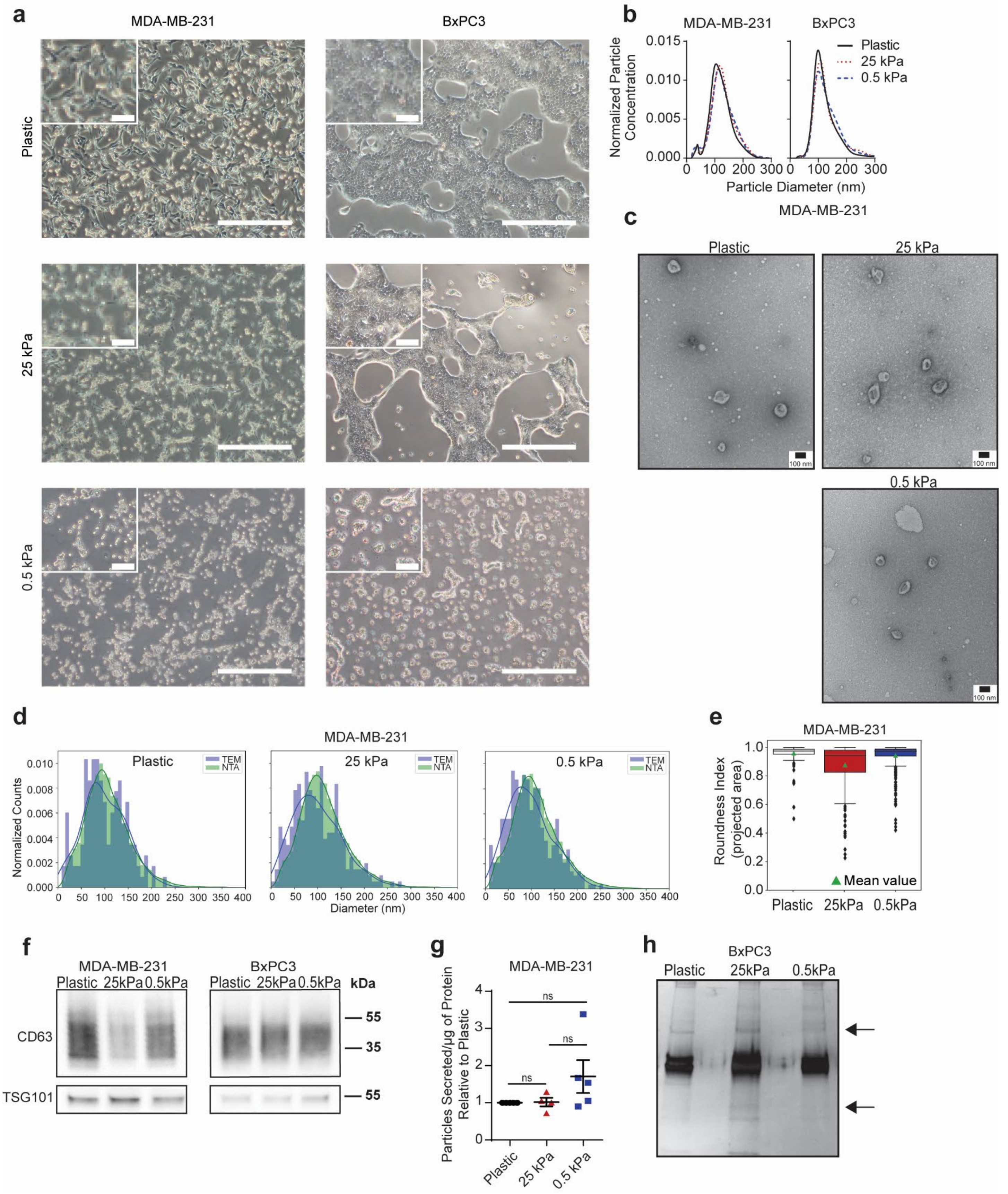

Figure S2: Isolation and characterization of EVs produced by cancer cells *in vitro.*

(a), Morphology of MDA-MB-231 human triple negative breast cancer cells and BxPC3 human pancreatic cancer cells cultured on tissue culture plastic, 25 kPa (stiffness of breast tumor tissue), and 0.5 kPa (stiffness of normal breast tissue) matrices. Scale bar, 500 μm. Inset scale bar, 100 μm. **(b)**, Size distribution of vesicles released by human triple negative breast cancer cells (MDA-MB-231) and human pancreatic cancer cells (BxPC3) on plastic dishes and on 25 kPa, and 0.5 kPa collagen I-coated matrices. **(c)**, Representative TEM images of EVs produced by MDA-MB-231 cells. Scale bar, 100 nm. Two biological repeats. **(d)**, Comparison of the size distribution of vesicles produced by MDA-MB-231 cells measured via NTA and machine-learning-based TEM. Two biological repeats. **(e)**, Roundness (projected area) index of EVs from TEM images. Two biological repeats. **(f)**, Representative western blots of EV markers CD63 and TSG101 for EVs produced by MDA-MB-231 and BxPC3 cells on plastic dishes and on 25 kPa and 0.5 kPa matrices. Nine biological repeats. **(g)**, Number of EVs released per microgram of protein relative to the plastic condition. Five biological repeats plastic EVs, four biological repeats stiff EVs, and five biological repeats soft EVs. **(h)**, Silver-stain of BxPC3 EV isolated proteins.
