# Supplementary material for: Small Extracellular Vesicles Promote Stiffness-mediated Metastasis
Source: Cancer Res Commun. 2024 May 9;4(5):1240–52. doi: 10.1158/2767-9764.CRC-23-0431 (PMC11080964; doi:10.1158/2767-9764.CRC-23-0431)
Supplement: Figure S3 — Comparison of protein cargo for plastic, stiff and soft EVs [file crc-23-0431-s06.pdf]

Figure S3: Protein cargo is different for plastic, stiff and soft EVs

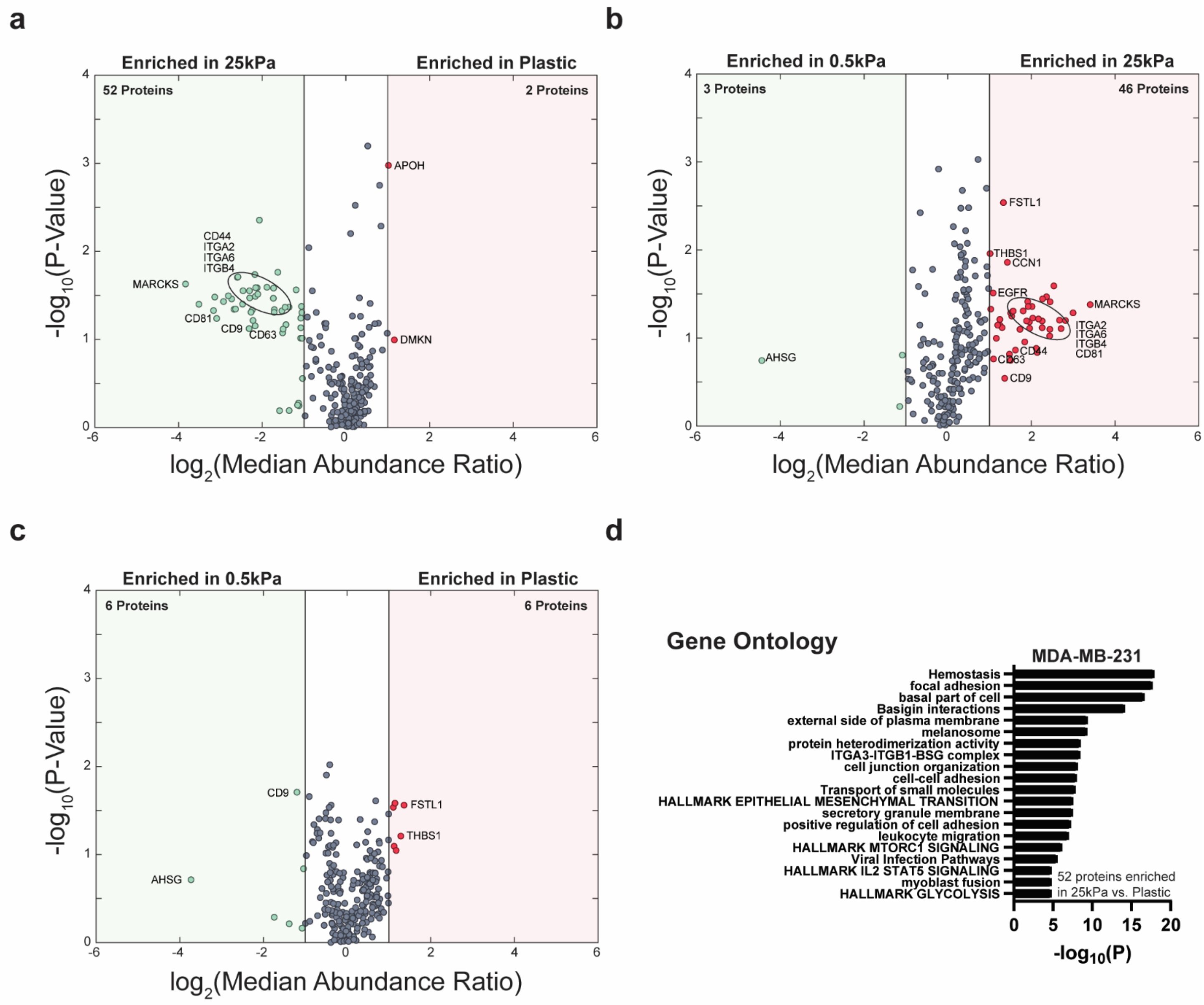

Figure S3: Protein cargo is different for plastic, stiff and soft EVs.

Volcano plots of differentially abundant proteins between (**a**) 25kPa and Plastic EVs, (**b**) 0.5kPa and 25kPa EVs, (**c**) 0.5kPa and Plastic EVs. The x-axis is the $\log_2$ ratio of the median abundance ratio between biological replicates. The y-axis is the $-\log_{10}$(P-value). Red denotes those proteins enriched >2-fold in the vesicle condition in the numerator. Green denotes those proteins enriched >2-fold in the vesicle condition in the denominator. The number of enriched proteins is written in each color region. Three biological repeats. **(d)** Gene ontology pathway analysis using Metascape for the 48 proteins enriched in stiff 25kPa EVs over the plastic EVs by MDA-MB-231 human breast cancer cells (Zhou *et al.*, 2019).
