# Supplementary material for: Small Extracellular Vesicles Promote Stiffness-mediated Metastasis
Source: Cancer Res Commun. 2024 May 9;4(5):1240–52. doi: 10.1158/2767-9764.CRC-23-0431 (PMC11080964; doi:10.1158/2767-9764.CRC-23-0431)
Supplement: Figure S4 — Plastic EVs have different biodistribution compared to stiff and soft EVs. [file crc-23-0431-s07.pdf]

Figure S4: Plastic EVs have different biodistribution compared to stiff and soft EVs

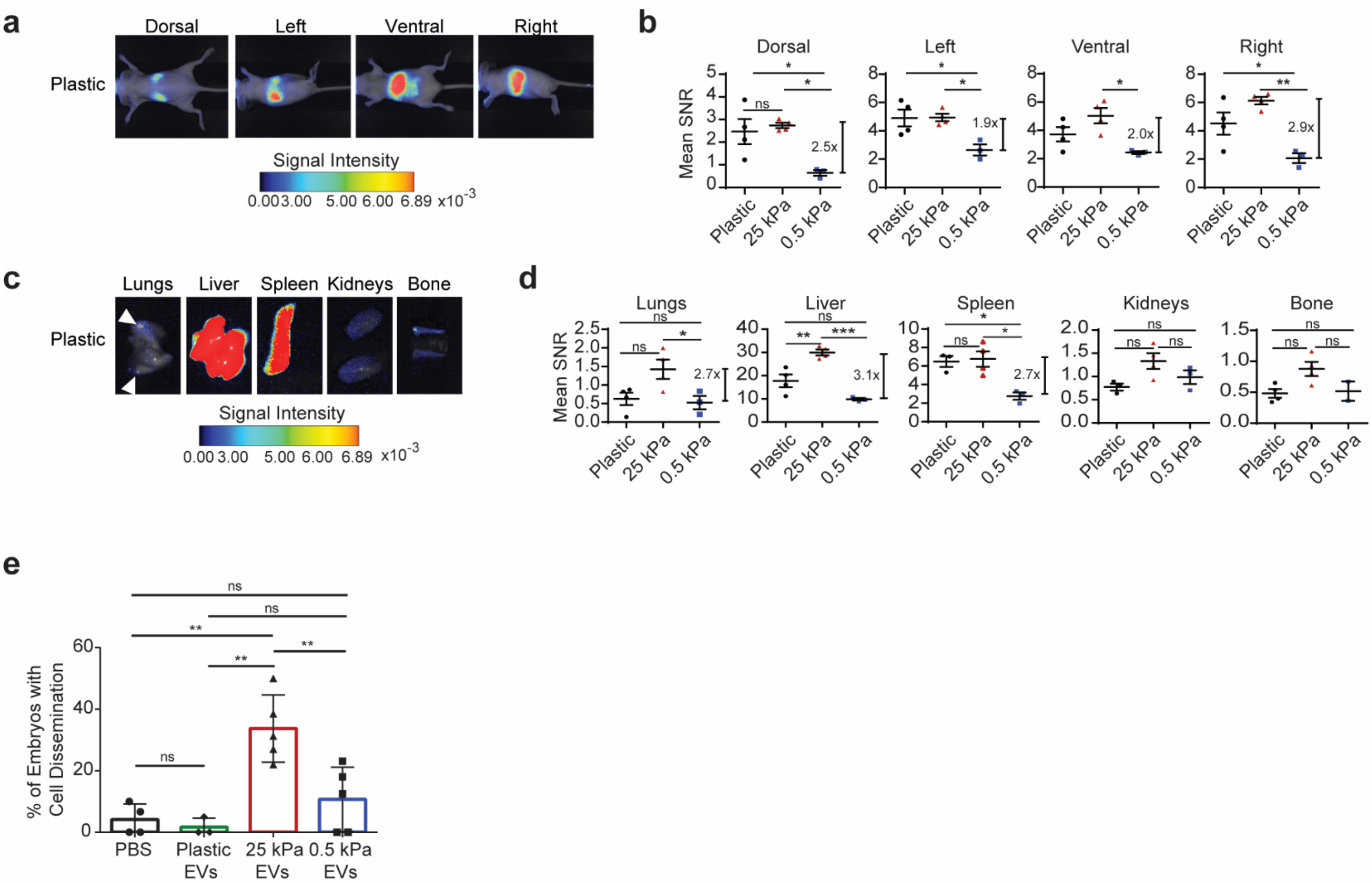


**Figure S4: Plastic EVs have different biodistribution compared to stiff and soft EVs.**

**(a)**, Near-infrared (NIR) imaging and **(b)**, mean-signal-to-noise ratio (SNR) of MDA-MB-231 vesicle biodistribution in dorsal, left, ventral and right sides (mean ± SEM). Signal intensity is in arbitrary units (a.u.). Three mice in 0.5 kPa condition, and four in plastic and 25 kPa conditions; one-way ANOVA. **(c)**, NIR imaging and **(d)**, mean SNR biodistribution in the lungs, liver, spleen, kidneys, and bone/bone marrow (mean ± SEM). Signal intensity is in arbitrary units (a.u.). Three mice in MDA-MB-231 0.5 kPa condition, and four in plastic and 25 kPa conditions; one-way ANOVA. Only three mice are shown for plastic in the spleen and kidneys plots since one mouse in plastic condition was missing images for the spleen and kidneys. **(e)**, Percentage of injected embryos with cancer-cell dissemination to the head or the tail. Total number of fish per condition is 47 for PBS control, 40 for plastic, 63 for 25 kPa, and 78 for 0.5 kPa condition. Three biological repeats of EVs for plastic and five for 25 kPa and 0.5 kPa EVs. One-way ANOVA.
